# Nuclear Import and Dimerization of Tomato ASR1, a Water Stress-Inducible Protein Exclusive to Plants

**DOI:** 10.1371/journal.pone.0041008

**Published:** 2012-08-10

**Authors:** Martiniano M. Ricardi, Francisco F. Guaimas, Rodrigo M. González, Hernán P. Burrieza, María P. López-Fernández, Elizabeth A. Jares-Erijman, José M. Estévez, Norberto D. Iusem

**Affiliations:** 1 Instituto de Fisiología, Biología Molecular y Neurociencias (IFIByNE-CONICET), Facultad de Ciencias Exactas y Naturales, Universidad de Buenos Aires, Buenos Aires, Argentina; 2 Departamento de Química Orgánica and CIHIDECAR, CONICET, Facultad de Ciencias Exactas y Naturales, Universidad de Buenos Aires, Buenos Aires, Argentina; 3 Departamento de Biodiversidad y Biología Experimental, Facultad de Ciencias Exactas y Naturales, Universidad de Buenos Aires, Buenos Aires, Argentina; 4 Laboratorio de Fisiología y Biología Molecular (LFBM), Departamento de Fisiología, Biología Molecular y Celular (FBMC), Facultad de Ciencias Exactas y Naturales, Universidad de Buenos Aires, Buenos Aires, Argentina; Indiana University School of Medicine, United States of America

## Abstract

The ASR (for ABA/water stress/ripening) protein family, first described in tomato as nuclear and involved in adaptation to dry climates, is widespread in the plant kingdom, including crops of high agronomic relevance. We show both nuclear and cytosolic localization for ASR1 (the most studied member of the family) in histological plant samples by immunodetection, typically found in small proteins readily diffusing through nuclear pores. Indeed, a nuclear localization was expected based on sorting prediction software, which also highlight a monopartite nuclear localization signal (NLS) in the primary sequence. However, here we prove that such an “NLS” of ASR1 from tomato is dispensable and non-functional, being the transport of the protein to the nucleus due to simple diffusion across nuclear pores. We attribute such a targeting deficiency to the misplacing in that cryptic NLS of two conserved contiguous lysine residues. Based on previous *in vitro* experiments regarding quaternary structure, we also carried out live cell imaging assays through confocal microscopy to explore dimer formation *in planta*. We found homodimers in both the cytosol and the nucleus and demonstrated that assembly of both subunits together can occur in the cytosol, giving rise to translocation of preformed dimers. The presence of dimers was further corroborated by means of in vivo crosslinking of nuclei followed by SDS-PAGE.

## Introduction

The ASR protein family has been first described in tomato [1] and is involved in water stress response and adaptation to dry environments [2]. It is widespread in the plant kingdom including numerous relevant crops, though absent in *Arabidopsis* [3]. This family has been classified as a new group within the Late Embryogenesis Abundant (LEA) superfamily [4]. *In vitro* experiments suggest that ASR1 protein can act as a chaperone preventing the unfolding of soluble cytosolic proteins under desiccation conditions [5]. Interestingly, ASR1 overexpression modified sugar content in potato [6]. Its grape homolog, VvMSA, is also associated with sugars [7] and has been described as a transcription factor that binds the enhancer of a hexose transporter gene from grape (VvHT1) [8]. This likely dual function, namely chaperone and transcription factor, is consistent with the observed double cellular localization of ASR1 in both cytosolic and nuclear fractions of tomato cells [9].

It has been demonstrated that recombinant ASR1 binds to DNA *in vitro* when present as a dimer [9]–[11]. *In vitro* experiments like atomic force microscopy and gel electrophoresis previously demonstrated that recombinant ASR1 readily forms homodimers that cannot be completely disaggregated *in vitro* by ionic detergents like SDS [11], similarly as the Alzheimer-causing amyloid protein [12].

Nuclear transport is generally performed by the cell through large protein nuclear pore complexes (NPCs) [13]. Their structure has been extensively studied and believed to be responsible for both passive and active nuclear import [14], processes that cannot be dissected with drugs since the available inhibitors act on both of them. In the case of active translocation, nuclear localization signals (NLSs) drive the shuttling of the proteins that possess them into the nucleus by binding to importin-like proteins through NPCs [15]. In the case of passive transport, it is generally accepted that proteins smaller than 50–60 kDa may enter the nucleus by simple diffusion through the NPC [15], [16] although some authors claim a bigger exclusion size when dealing with GFP fusion proteins [17]. Nuclear localization of ASR1, the member of the family most studied at the structural level, has been determined [1], [9] and also expected based on sorting prediction softwares developed later [18]–[20]. A monopartite NLS [21] can be found in the primary sequence which suggests active import, but on the other hand, ASR1 protein (12.5 kDa) is small enough for passive transport.

## Methods

### Plasmid Constructs

pCambia 1302, 1303 and 1304 vectors [22] were used to create GFP:His6 (GFP), GUS:GFP:His6 (GUS:GFP) and GFP:GUS:His6 (GFP:GUS) fusion proteins. All the inserts for subcellular localization experiments were made by PCR and restriction cloning using the primers indicated in **Table S1**. For BiFC constructs, we first made the fusions with EYFP halves by PCR and restriction cloning (**Table S1**) and then subcloned the BiFC expression cassette into pCambia 1300 [22] by PCR and restriction enzyme cloning. For the additional BiFC construction, ASR1:GUS:vNLS was first constructed by running two consecutive PCRs, using ASR1 cloned into pCambia1303 as template and the primers indicated in **Table S1**. After that, ASR1; ASR1:GUS and ASR1:GUS:vNLS were cloned into the gateway system (by PCR amplification and BP reaction) and subcloned into their respective destination vectors by LR recombination. For immunolocalization, ASR1 was subcloned into pGWB2 vector [23] by LR recombination. All constructs, except for the gateway expression clones, were sequenced by Macrogen Korea. Molecular weights of all encoding proteins are listed in **Table S2**.

### Immunolocalization

Sections of *Nicotiana benthamiana* leaves were fixed for 2 h at 4°C using a mixture of 4% para-formaldehyde and 0.05% glutaraldehyde in 0.1 M saline phosphate buffer (PBS) pH 7.2, rinsed with PBS for 30 min, dehydrated in a graded ethanol series with changes every 30 min and embedded in LRW resin (Polyscience, Inc.; 17411) as previously described [24]. Cross sections (1 µm thick) were obtained with an ultramicrotome (Reichert-Jung, Vienna, Austria) using a diamond knife and processed for immunofluorescence. After rehydration in PBST (0.05% Tween 20 in 10 mM PBS, pH 7.2), sections were pretreated 1 h with 1% BSA in PBST and incubated overnight with rabbit anti-ASR1 antiserum or mouse anti-MYC (Santa Cruz) antibody diluted 1∶50 and 1∶100, respectively, in PBS containing 0.1% BSA. After five washes in PBST, sections were incubated for 1 h with the secondary antibodies Alexa-Fluor-488 anti- rabbit and Alexa-Fluor-488 anti-mouse IgG (Invitrogen) diluted 1∶1,000 in PBS supplemented with 0.1% (w/v) BSA. After additional rinses in PBS plus 0.05% Tween 20, sections were examined using a laser a scanning confocal microscope.

### *Agrobacterium* Cultures and Infiltration

*Agrobacterium tumefaciens* cells (strain DB31.01) carrying the different constructs were grown on 10 ml of LB supplemented with gentamycin, rifampycin and the appropriate selection antibiotic overnight. The culture was pelleted, resuspended in 10 ml of 10 mM MgCl_2_ in the presence of 150 µg/ml acetosiryngone and incubated 3 hours at 4°C. Young leaves of 3–4 week-old *Nicotiana benthamiana* plants were infiltrated with a 0.1 O.D. culture of *Agrobacterium tumefaciens* harboring the different constructs. For BiFC experiments, leaves were co-infiltrated with a 0.5 O.D. culture of an equal mix of both used cultures. After 3 days, the same leaves were infiltrated with a 10 µg/ml of water dissolved DAPI, incubated in the dark for 20 minutes and observed in a confocal microscope.

### Microscopy Settings

Images were acquired in an Olympus FV1000 confocal microscope (Olympus Latin America) with a 60× 1.2 N.A. water immersion objective. For excitation, we used 405 and 488 lasers for DAPI and Alexa488/GFP/Venus/EYFP, respectively. Emission filters were 430–470 nm for DAPI and 505–525 nm for Alexa488/GFP/Venus/EYFP. Images were acquired in a sequential mode.

### Co-localization Analysis

Since it was not possible to wash non-bound DAPI from the infiltrated leaves, the DAPI signal was processed and the non-nuclear signal (mostly on the cell wall) was manually removed. Pearson’s coefficients of 128×128-pixel areas comprising the nucleus were calculated using the Jacop macro available for ImageJ [25].

### Nuclear Protein Extractions

10 g fruit pericarp tissue was frozen with liquid nitrogen and ground to a fine powder with mortar and pestle. Nuclei were purified as previously described [26] except for the crosslinking step, performed after tissue grinding. Untagged ASR1 protein from nuclei was further purified using a Ni^2+^-sepharose column (His-trap HP, GE Healthcare). Proteins eluted from the column were run in SDS-PAGE, transferred into a nitrocellulose membrane and immunoblotted with rabbit anti-ASR1 antiserum.

## Results

### Subcellular Localization of ASR1 and Functionality of its Putative NLS

**Figure 1:**
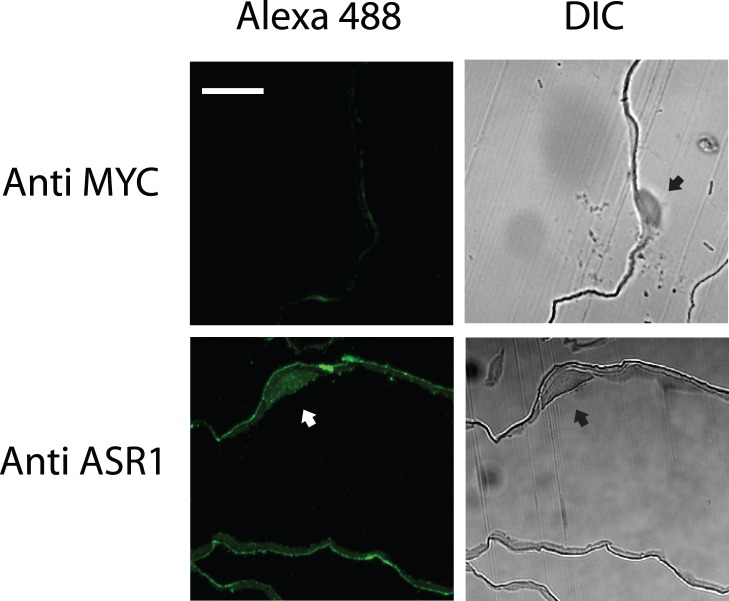
Both nuclear and cytosolic ASR1 immuno-localization. Cross sections of *N. Benthamiana* leaves expressing untagged tomato ASR1 were probed with anti-ASR1 or non-specific anti-MYC antibodies. Alexa 488 signal (see Methods) showed dim fluorescence in the anti-MYC control while samples probed for ASR1 showed both nuclear (arrows) and cytosolic localization. Nuclei can be also observed in DIC images. Scale bar  =  10 µm.

We first determined subcellular localization of ASR1 in histological plant samples by immunodetection. Untagged tomato ASR1 localized in both nucleus and cytoplasm when transiently expressed in *Nicothiana benthamiana* leaves (**Figure 1**), as typically found in small proteins readily diffusing through nuclear pores. This result prompted us to run nuclear prediction programs for several ASR1 homologs, which highlighted a carboxy-terminal NLSs in most of them (**Table 1**). NUCLEO program showed high probability of nuclear localization for most of the proteins except for the citrus ASR homolog [20]. Similarly, YLOC [19] showed high probability and high confidence of nuclear localization for all the orthologous polypeptides. Consistently, Euk-mPLoc 2.0 [18] predicted all the proteins to be nuclear (data not shown). Alignments of the NLSs showed that tomato ASR1 has two lysine residues, very close to the carboxy terminus, in a position different from that of LLA23 and VvMSA, whose nuclear import signals were previously proved to be functional [7], [27].

**Table 1:** Features of several “NLSs” from ASR1 homologues.

Species	Yloc		Predicted NLS	Observed	NLS aligned sequences	MW (kDa)
	Prob	Conf				
Tomato	99.72	0.87	0.88	Nuc/Cyto	GGFAFHEHHEKKDAKKEEKKAEGGHHHLF	12.5
Potato	99.72	0.87	0.86	–	GGFALHEHHEKKDAKKEQKKAEGGHHHLF	12.5
*N. Benthamiana*	97.39	0.69	0.67	–	GGFAFHEHHDKKDAKKEQKEAEGGKHHHLF	11.1
Grape	73.43	0.62	0.86	Nuc	GGFAFHEHHEKKEAKEEDEEAHG∼KKHHHLF	16.7
Lily	97.15	0.90	0.66	Nuc	GGYTFHEHHEKKTLKKENEEVEG∼KKHHFFG	16.1
Maize	92.09	0.74	0.61	–	GGFAFHEHHEKKKDHKDAEEAGGEKKHHFFG	15.7
Rice	87.07	0.67	0.54	–	GGYAFHEHHEKKKDHKSAEESTGEKKHHLFG	15.4
Citrus	87.18	0.14	0.40	–	GGFAFHEHHEKKEAKEEDQEAHG∼KKSTTISFN	10.8

NLSs of ASR homologues from different species were compared to that of tomato ASR1. NUCLEO software [20] renders high probability of nuclear localization (“predicted NLS” column) for most of the polypeptides. Similarly, YLOC [19] predicts high probability and high confidence of nuclear localization for all the orthologous proteins except for citrus. Species with experimentally proven nuclear localization for ASR are indicated in the “observed” column. Alignment of all NLSs reveals differences in the carboxy-terminal region at the lysine (K) positions highlighted in gray. Molecular weight (MW) of full length mature polypeptides is shown.

**Figure 2:**
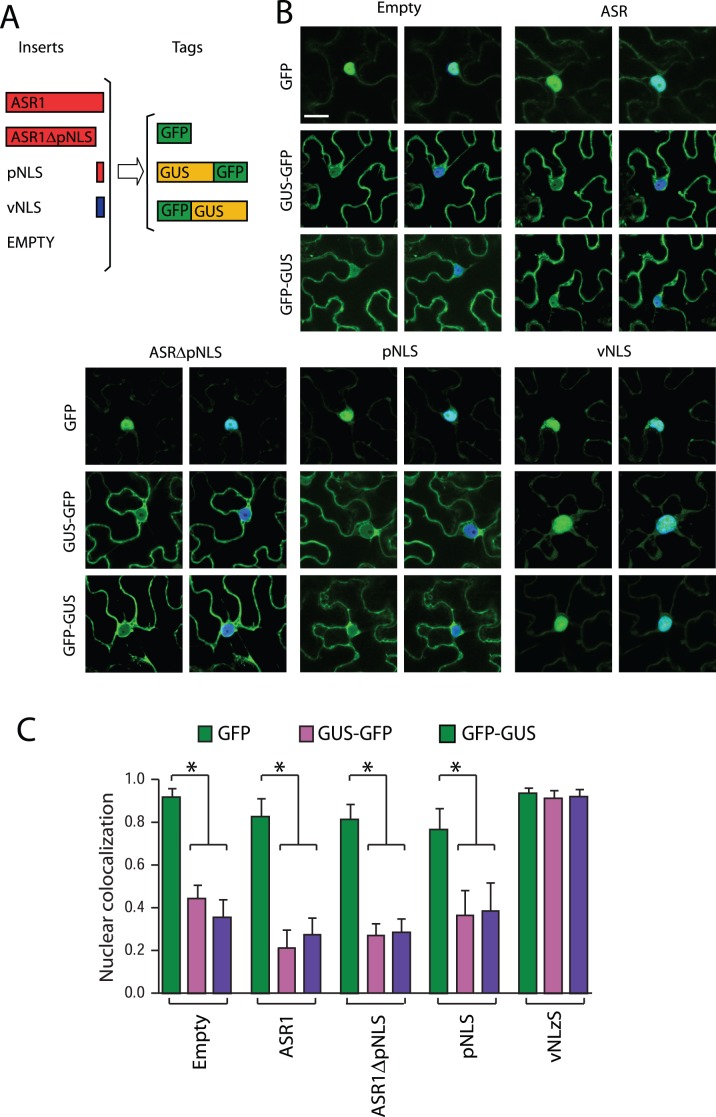
Nuclear localization of ASR1 by passive diffusion and not determined by NLS. A) Set of constructs used to address the functionality of the putative NLS (pNLS) of ASR1. B) Nuclear localization measured through co-localization of GFP signal (in green) with DAPI nuclear staining (in blue) by transient expression in *N. Benthamiana* leaves. Representative images show two clear different localization patterns. On the left of each panel, GFP channel (green). On the right, GFP channel merged with DAPI signal (blue). Scale bar  =  10 µm. C) Pearson’s coefficients of co-localization with DAPI signal of empty vectors, full length ASR1, truncated ASR1 without pNLS signal (ASR1?NLS), ASR1 pNLS and viral nuclear localization signal (vNLS) fused to either GFP or GFP-GUS were calculated. * P<0.0001, n = 15.

To test the functionality of the putative NLS (pNLS) from tomato ASR1, we designed several GFP- or GUS:GFP-tagged constructs containing either full length ASR1 or ASR1 devoid of its own pNLS, in addition to the pNLS alone and an authentic NLS from viral origin (vNLS) as control [16] (**Figure 2A**). Controls with empty vectors showed a dual nuclear/cytosolic distribution for GFP (28.4 kDa), similar to the one observed in the immunolocalization experiment (**Figure 1**), and an almost exclusively cytosolic localization for GFP-GUS (96.9 kDa) due to its high molecular weight as previously described for epidermal cells from *Nicothiana benthamiana* leaves [16]. All the ASR1 constructs showed the same pattern as the empty vector controls, even when attaching pNLS from ASR1. Only the functional vNLS was able to target most of the GUS:GFP or GFP:GUS fluorescence to the nucleus (**Figure 2B**). Pearson coefficient showing co-localization with DAPI was the same as in the empty vectors for all the constructs except for the viral NLS controls fused to GFP:GUS or GUS:GFP (**Figure 2C**). We thus conclude that the previously so-called “pNLS” is dispensable and therefore, the observed nuclear localization of native ASR1 can be attributed to a simple diffusion event rather than to an active translocation process.

### *In planta* Homodimer Formation

**Figure 3:**
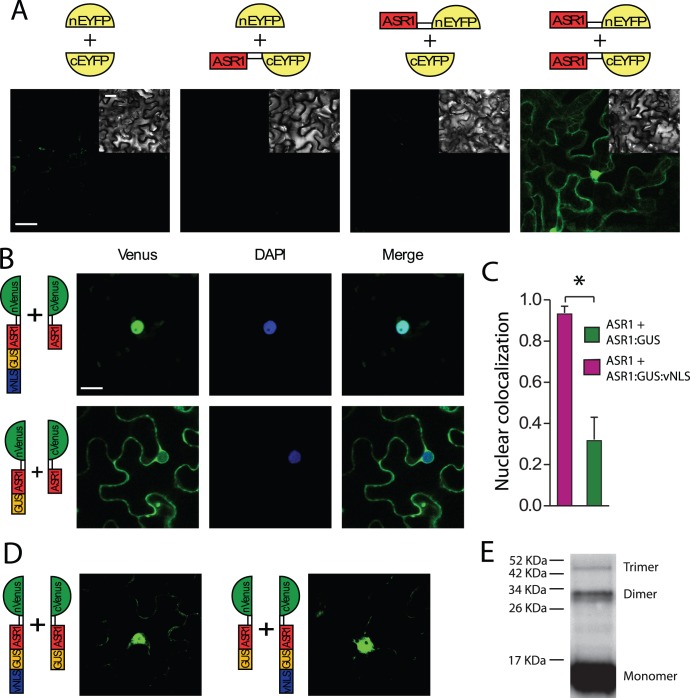
*In vivo* ASR1 homodimer formation. A) Bimolecular Fluorescence Complementation (BiFC) experiments with transiently expressed ASR1 fusion proteins were able to reconstitute the two halves of EYFP. *N. Benthamiana* leaves were infiltrated with the four possible combinations resulting from one nEYFP (empty and ASR1-fused) and one cEYFP (empty and ASR1-fused) construct. Scale bar  =  10 µm. B) Further BiFC experiments were designed using ASR1:nVenus as a partner of ASR1:GUS:cVenus or ASR1:GUS:vNLS-cVenus chimerical proteins. Fluorescence was detected only in nuclei for the ASR1:GUS:vNLS:cVenus chimera and mostly in cytosol for the ASR1-GUS-cVenus construct. Venus (left, in green), DAPI (middle, in blue) and merged images (right) are shown. Scale bar  =  10 µm. C) Nuclear localization was measured through co-localization of GFP signal with DAPI nuclear staining (blue). Pearson’s coefficients of both construct combinations were calculated. * P<0.005, n = 15. D) Control experiments were performed with ASR1:GUS and ASR1:GUS:vNLS fused to nVenus and cVenus and viceversa. An unexpected signal was observed mostly in the nucleus. E) Ni^2+^-purified ASR1 obtained from crosslinked tomato fruit nucleus showed dimers and even trimers when run in SDS-PAGE and probed with anti-ASR1 antibody. Molecular weight markers are indicated.

We addressed the question whether the observed stable ASR1 dimers are formed only after protein extraction *in vi*tro or, alternatively, already present *in planta*. For that purpose, we tested dimerization by Bimolecular Fluorescence Complementation (BiFC) using the non-fluorescent carboxy- and amino-terminal halves of the Enhanced Yellow Fluorescent Protein (EYFP), each fused to ASR1. Fluorescence turned out to be evident only when both ASR1-fused EYFP halves were supplied to the cells, thus proving homodimerzation *in vivo*. Surprisingly, homodimers were observed in both nucleus and cytosol (**Figure 3A**). Due to its small size, 52.2 kDa, it is plausible for these dimers to enter the nucleus by simple diffusion.

### Intracellular Assembly of Homodimers

In order to assess the intracellular compartment for ASR1 dimer formation and the possibility of a functional pNLS only in homodimers, we performed another BiFC assay using split Venus fluorescent protein [28]. For this experiment, we generated two constructs: 1) ASR1:GUS, which, due to its large size, remains in the cytosol; and 2) ASR1:GUS:vNLS, targeted to the nucleus. Both constructs were in turn fused to the carboxy-terminal half of Venus fluorescent protein (10.6 kDa) (**Figure 3B**). As a dimer partner for both polypeptides, we added ASR1 fused to the small sized (20.9 kDa) amino-terminal half of Venus. When ASR1:GUS was used, ASR1 dimers were observed mostly in the cytosol (**Figure 3B-C**), confirming that dimerization of native ASR1 can occur in that compartment. Moreover, we validated that pNLS of ASR1 is non-functional, even in the context of larger dimers. Besides, when one monomer was forced to move to the nucleus (because of the attaching of GUS with an active viral NLS), fluorescence was seen only in the nucleus, suggesting that dimers could assemble there as well (**Figure 3B-C**).

Since nuclear proteins are synthesized in the cytosol and thought to be immediately translocated to the nucleus, it is also possible for dimers, in principle, to be assembled before translocation. To test this alternative scenario, we performed a BiFC assay with the monomers ASR1:GUS:vNLS (targeted to the nucleus) and ASR:GUS (unable to go to the nucleus alone) together, fused to the carboxy- and amino-terminal halves of Venus, respectively, and viceversa (**Figure 3D**). As a clear signal was observed mainly inside the nucleus (**Figure 3D**), a mechanism that poses translocating dimers is possible (**Figure S1**). Since BiFC fluorophores are stable once formed [29], it is still possible for these artificial dimers to be formed in the cytosol and then translocated to the nucleus where they would be unable to dissociate. To validate these results for the native polypeptide, we further corroborated the presence *in planta* of endogenous ASR1 dimers by means of formaldehyde-crosslinking. For this purpose, we used tomato fruits which naturally present high ASR1 expression. After crosslinking, nuclear proteins were passed through an affinity chromatography (Ni^2+^-agarose) column, run into SDS-PAGE and immunoblotted with anti-ASR1 antibodies. We could observe not only the presence of dimers but also trimers. Quantification of the obtained protein bands indicates that more than 90% of nuclear ASR1 is present in a monomeric form (**Figure 3E**).

## Discussion

LLA23 and VvMSA (lily and grape ASR proteins) are actively imported to the nucleus by their respective NLSs [7], [27]. In contrast, tomato ASR1 behaves in a different way as it is translocated in a NLS-independent manner. It is remarkable that small proteins like LLA23 and VvMSA cannot enter the nucleus without their NLS despite of being smaller than 17 kDa [7], [27], whereas tomato ASR1 (12.5 kDa) can. This apparent contradiction may be explained because of the use of different plant species for the localization experiments. It is possible that the nucleus pore architecture in *Nicothiana benthamiana* differs from that in *Chenopodium quinoa* or tobacco cell line BY2, which has been used as experimental models for LLA23 and VvMSA localization studies [7], [27]. Similarly, tomato ASR1 localized mainly in the cytosol when expressed in yeast [30]. It could be interesting to test whether these ASR1 homologs still need their NLS to enter the nucleus of *N. benthamiana* leaf epidermal cells. On the other hand, the NLS from these polypeptides contains two lysine residues in a different position when compared to the tomato ortholog. Therefore, it is tempting to speculate that the occurrence of such two neighbouring lysines in that precise position is a requisite for active nuclear targeting, at least in ASR-like proteins. In this context, it would be interesting to test the intracellular localization of the ASR-like protein from our experimental model *Nicotiana benthamiana*, which exhibits only one lysine residue at that position in the primary sequence. Further assays by means of constructs containing these two lysines from grape and lily NLS should be performed to prove if the lack of these residues is actually responsible for the observed behaviour regarding passive transport.

**Figure 4:**
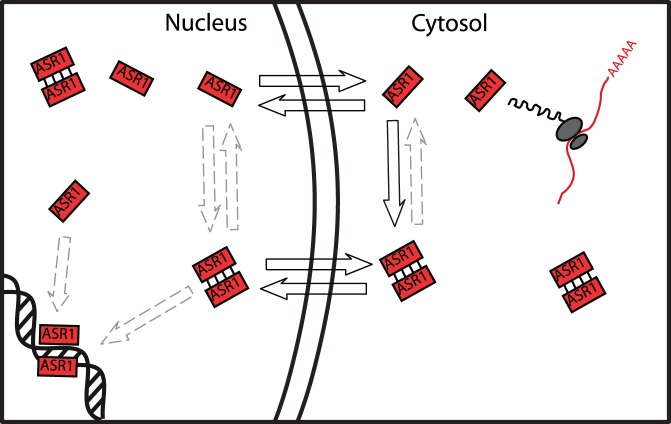
Model for tomato ASR1 dimerization and translocation. ASR1 monomers and dimers may enter or leave the nucleus by simple diffusion, in an NLS- independent manner. While dimer formation in cytosol has been demonstrated, ASR1 dimers in the nuclei derive from preformed cytosolic dimers or might be also formed there. ASR1 might bind DNA as a monomer or dimers for target gene regulation. Solid line arrows indicate proven events; dashed line arrows, events yet to be demonstrated *in vivo*.

Several reports describe different relationships between dimerization and nuclear translocation of proteins. For example, STAT1 translocates to the nucleus after dimerization upon the formation of a complete NLS in the cytosol triggered by phosphorylation [31]. Similarly, STAT3 enters the nucleus as a dimer [32]. In contrast, protein ERK enters nuclei as a monomer, also triggered by phosphorylation http://www.jbc.org/search?author1=JulieWilsbacher&sortspec=date&submit=Submit [33]. In contrast to STAT1, artificial high molecular weight ASR1 homodimers are unable to translocate, indicating that an active NLS is not formed upon dimerization. Taken together, results lead us to conclude that ASR1 dimers can reach the nucleus by two traffic alternatives: either as homodimers preformed in the cytosol or as translocating monomers to be later dimerized (**Figure 4**). From a kinetics point of view, data are consistent with ASR1 dimers assembled in the cytosol more rapidly than the active nuclear import event driven by vNLS used in Bifc experiments. Nevertheless, dimerization could also occur in the nucleus, perhaps facilitated by DNA binding as previously suggested [9], [11]. Finally, it has not yet been demonstrated that ASR1 or any other orthologous protein is present *in planta* in a monomeric state in equilibrium with a dimeric form. In this context, to our surprise, crosslinked nuclear extracts from tomato fruits revealed that most of ASR1 is present as a monomer in the nucleus co-existing with dimers and even trimers. A plausible scenario can be envisaged posing ASR1 dimers as chromatin-bound [11] and monomers involved in chaperon-like functions [5].

## Supporting Information

Figure S1 **Proposed explanation for Bifc results.** This cartoon is consistent with the observed results depicted in Figures 2 and 3. Nuclear dimers can appear as a consequence of either nuclear dimerization of translocating monomers or translocation of preformed (in the cytosol) dimers, mechanisms that are not mutually exclusive.
(EPS)

Table S1 **Primers and plasmids**.** First sheet: primers designed to generate the different inserts. Restriction enzymes sites and Gateway sites within the primers are listed. Second sheet: list of plasmid vectors used to subclone the different inserts. Cloning strategies to generate the final constructs are also indicated.**
(XLS)

Table S2 **Molecular weight of polypeptides.** Molecular weight of the polypeptides encoded by the inserts of the constructs used throughout the work.
(XLS)
